# Correlation between the triglyceride-glucose index and the onset of atrial fibrillation in patients with non-alcoholic fatty liver disease

**DOI:** 10.1186/s13098-023-01012-1

**Published:** 2023-05-09

**Authors:** Yao Zhang, Leigang Wang, Jiaxin Qi, Bing Yu, Jianqi Zhao, Lin Pang, Wenjing Zhang, Liang Bin

**Affiliations:** 1 Shanxi Medical University, Taiyuan, 030000 Shanxi China; 2 Department of Cardiovascular Medicine, Second Hospital of Shanxi Medical University, 382 Wuyi Road, Taiyuan, 030000 Shanxi China

**Keywords:** Triglyceride-glucose index, Insulin resistance, Non-alcoholic fatty liver disease, Atrial fibrillation, Arrhythmia

## Abstract

**Background:**

Non-alcoholic fatty liver disease (NAFLD) is associated with atrial fibrillation (AF). Insulin resistance (IR) is the main cause of the high prevalence of AF in NAFLD patients. The triglyceride-glucose index (TyG) is a novel IR-related indicator implicated in the incidence and severity of NAFLD. However, the role of TyG in determining the risk for AF in patients with NAFLD remains unclear.

**Methods:**

A retrospective study was conducted on 912 patients diagnosed with NAFLD via ultrasonography. These patients were divided into two groups: (1) NAFLD+ AF and (2) NAFLD+ non-AF. Least Absolute Shrinkage and Selection Operator (LASSO) regression was used to assess the correlation between the TyG index and the high risk for AF. A receiver operating characteristic (ROC) curve was constructed to evaluate the predictive value for the TyG index for AF. Restricted cubic splines (RCS) were used to test the linear correlation between TyG and the risk for AF.

**Results:**

A total of 204 patients with AF and 708 patients without AF were included in this study. The LASSO logistic regression analysis showed that TyG was an independent risk factor for AF (odds ratio [OR] = 4.84, 95% confidence interval [CI] 2.98–7.88, P < 0.001). The RCS showed that the risk for AF increased linearly with TyG over the entire TyG range; this risk was also evident when the patients were analyzed based on sex (P for nonlinear > 0.05). In addition, the correlation between TyG and AF was a consistent finding in subgroup analysis. Furthermore, ROC curve analysis showed that TyG levels combined with traditional risk factors improved the predictive value for atrial fibrillation.

**Conclusion:**

The TyG index is useful in assessing the risk for atrial fibrillation in patients with NAFLD. Patients with NAFLD and increased TyG indices have higher risks for atrial fibrillation. Therefore, TyG indices should be assessed when managing patients with NAFLD.

**Supplementary Information:**

The online version contains supplementary material available at 10.1186/s13098-023-01012-1.

## Introduction

Atrial fibrillation (AF) is the most common arrhythmia observed in clinical practice, with a prevalence of approximately 1 to 4%. Even so, the prevalence and incidence of AF are steadily increasing [1]. Patients with a history of AF have an increased risk for an embolic stroke, heart failure, myocardial infarction, dementia, and chronic kidney disease. Therefore, AF is a significant burden for cardiovascular health worldwide [1, 2].

Non-alcoholic fatty liver disease (NAFLD) is the most common liver and metabolic disease worldwide. Recent studies have shown that patients with NAFLD have an increased risk of arrhythmias, including AF and ventricular arrhythmias [3, 4]. Therefore, in specific populations (such as patients with NAFLD), techniques that enable the early detection of AF are very important. Currently, AF is detected using an electrocardiogram (ECG). ECGs are extremely limited in the early detection of AFs. Therefore, alternate techniques are needed to facilitate the early detection of AFs.

Insulin resistance (IR) is an integral part of the pathogenesis of NALFD [5]. IR is implicated in the pathogenesis of AF [6]. Studies have shown that systemic IR caused by NAFLD promotes epicardial fat accumulation. Furthermore, NAFLD-related changes may induce cardiac structure, electrical conduction, and autonomic remodeling. Therefore, NAFLD-related changes increase the likelihood of arrhythmias [7]. Clinical studies have also revealed a similar correlation between IR and AF. A significant correlation between IR and the development of AF, independent of other known risk factors, including obesity, was identified in a previous study [8] using a homeostatic model to assess the degree of IR (HOMA-IR).

The triglyceride–glucose index (TyG), a novel IR-related index, strongly correlates with HOMA-IR and hyperinsulinemia-euglycemic clamp results [9, 10]. TyG has a stable role in identifying and predicting multiple diseases [11–13]. Previous studies have shown that the TyG index correlates with the incidence and severity of NAFLD [14, 15]. Nonetheless, the relationship between the TyG index and the incidence of AF in patients remains unclear. Therefore, this study aimed to determine the correlation between the TyG index and the incidence of AF in patients with NAFLD.

## Methods

### Research population

In this study, 912 patients who underwent abdominal ultrasonography (US) and were hospitalized at the Department of Cardiology, Second Hospital of Shanxi Medical University, between July 2021 and July 2022, were retrospectively analyzed. The exclusion criteria were as follows: valvular heart disease or prosthetic valve implantation, ablation of atrial fibrillation, history of alcohol consumption, secondary liver disease (e.g., viral hepatitis or autoimmune liver disease), structural heart disease (e.g., rheumatic heart disease or cardiomyopathy), kidney disease or end-stage renal disease (ESRD), infectious disease, acute infection or malignant tumor history within the last 2 weeks, and incomplete medical records. Finally, 204 patients with nonvalvular AF (127 patients with paroxysmal AF and 77 patients with persistent AF) and 708 patients without AF (sinus rhythm) were included in the study. The study was approved by the Ethics Committee of our hospital and was conducted in accordance with the principles of the Declaration of Helsinki. Due to the retrospective nature of the analysis, the requirement for informed consent was waived.

AF was determined through [16] (1) ECG reports (defined as standard 12-lead ECG recordings showing a ≥ 30-s rhythm of the heart, undiscernible repetitive P-waves, and irregular RR intermittent diagnosis of AF) and (2) patients self-reporting histories of AF with clear physical evidence.

NAFLD was diagnosed [17] based on the criteria recommended by the Chinese Society of Liver Diseases, ultrasound findings of fatty liver, and exclusion of other causes of chronic liver disease. Fatty liver disease was diagnosed in patients with at least two of the following three findings: (1) a diffuse enhancement of the liver near-field echo that was stronger than that of the kidney, (2) a poorly delineated structure of the intrahepatic bile duct, and (3) a gradual attenuation of the far-field echo of the liver.

### Clinical and laboratory data

Anthropometric data and parameters including sex, age, height, weight, systolic blood pressure (SBP), diastolic blood pressure (DBP), hypertension (HTN), diabetes mellitus (DM), coronary heart disease (CHD), smoking history, and medication use were collected from electronic medical records. Overnight fasting blood samples were obtained to analyze biochemical variables, including alanine aminotransferase (ALT), aspartate aminotransferase (AST), serum creatinine (Scr), total cholesterol (TC), triglycerides (TG), high-density lipoprotein cholesterol (HDL-C), low-density lipoprotein cholesterol (LDL-C), fasting blood glucose (FBG), and other biochemical indicators. The body mass index (BMI) and TyG indices were also calculated. BMI was calculated as the weight (in kilograms) per height squared (in meters), while the TyG index was calculated using the formula ln [TG (mg/dL) × FBG (mg/dL)/2].

### Statistical analysis

Statistical analyses were performed using the SPSS version 26.0 software and R version 4.0.1. Continuous variables were expressed as mean ± standard deviation (SD) or median (interquartile range, IQR). Categorical variables were expressed as percentages. The presence and absence of AF were comparatively analyzed in patients. Continuous variables were compared using an independent sample t-test or Mann–Whitney U test. Categorical variables were compared using the Chi-square test or Fisher's exact test. P values were selected based on the results of the homogeneity test of variance. All the variables (including sex, age, BMI, systolic blood pressure, diastolic blood pressure, hypertension, diabetes, coronary heart disease, smoking history, medication history, ALT, AST, Scr, TC, TG, HDL-C, LDL-C, FBG, and TyG) between the two groups were included in the least absolute shrinkage and selection operator (LASSO) regression analysis, with AF as the outcome variable. The regularization parameter λ corresponding to one standard deviation from the minimum mean square error (Mean-Squared-Error) was selected as the most appropriate by cross-validation λ and select indicators with non-zero coefficients for multi-factor binary logistic regression analysis. Receiver operating characteristic (ROC) curves were constructed to evaluate the predictive value of the TyG index for AF. The trend Chi-square test was used to analyze multiple groups of categorical variables. The Kruskal–Wallis test was used to analyze multiple groups of continuous variables. The correlation between the TyG index and AF was evaluated using restricted cubic splines (RCS) with three nodes (10th, 50th, and 90th percentiles). Subgroup analyses were conducted, according to sex, age, BMI, smoking, HTN, diabetes, and CHD, to test the stability of the correlation between the TyG index and AF. Statistical significance was set at P < 0.05 (bilateral).

## Results

### Baseline characteristics in the AF and non-AF group

**Fig. 1:**
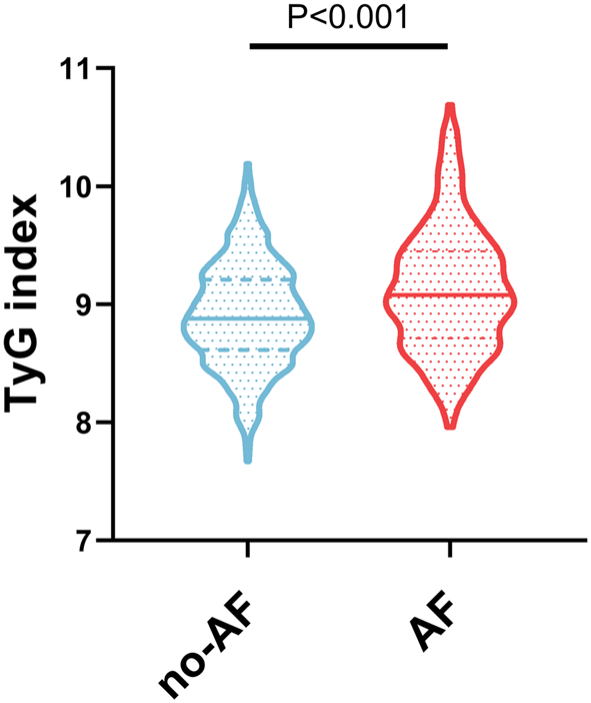
TyG level of patients in the atrial fibrillation (AF) group and non-AF group

Data were collected from 912 patients with NAFLD (561 men and 351 women), including 204 patients with AF and 708 without. The patients had a mean age of 68.78 ± 11.15 and 56.25 ± 10.31 years in the NAFLD with and without AF groups, respectively. Table 1 shows the demographic and clinical characteristics of the groups. There were no significant differences in sex, smoking history, DM, medication use, DBP, ALT, and AST between the groups. The NAFLD with AF group had higher groups of patients with a history of smoking, HTN, DM, higher mean participant ages, higher mean BMI, SBP, Scr, TG, FBG, and TyG levels; and lower HDL-C and LDL-C levels (all P < 0.05) (Fig. 1).

**Table 1:** Demographic and clinical characteristics of participants according to the presence of AF

	AF (204)	Non-AF (708)	P
Age (years)	68.78 ± 11.15	56.25 ± 10.31	< 0.001
Gender (male)	132 (64.7)	429 (60.6)	0.287
Smoke [(n%)]	76 (37.3)	305 (43.1)	0.137
HT [(n%)]	149 (73.0)	382 (54.0)	< 0.001
DM [(n%)]	64 (31.4)	201 (28.4)	0.408
CHD [(n%)]	121 (59.3)	352 (49.7)	0.016
BMI (kg/m^2^)	26.47 ± 2.95	25.89 ± 2.75	0.013
SBP (mmHg)	136.36 ± 20.15	130.84 ± 18.27	< 0.001
DBP (mmHg)	80.09 ± 14.14	79.99 ± 12.76	0.934
ALT (U/L)	23.20 (17.25,29.18)	24.35 (16.83,33.00)	0.197
AST (U/L)	22.30 (18.50,27.60)	22.70 (18.20,28.80)	0.540
Scr (mmol/L)	71.00 (63.00,82.00)	66.00 (56.00,75.00)	< 0.001
TC (mmol/L)	3.71 (3.10,4.50)	4.43 (3.66,5.23)	< 0.001
TG (mmol/L)	1.81 (1.27,2.39)	1.60 (1.24,2.09)	0.008
HDL-C (mmol/L)	1.02 (0.90,1.19)	1.10 (0.94,1.29)	< 0.001
LDL-C (mmol/L)	2.02 (1.49,2.57)	2.38 (1.91,2.87)	< 0.001
FBG (mmol/L)	5.89 (5.24,7.65)	5.50 (4.89,6.47)	< 0.001
Anti-diabetic drugs[(n%)]	55 (27.0)	181 (25.6)	0.688
Lipid-lowering drugs[(n%)]	73 (35.8)	205 (29.1)	0.062
TyG	9.12 ± 0.53	8.01 ± 0.44	< 0.001

Values are expressed as mean ± standard deviation, no. (%), or median (interquartile range). P < 0.05 (two-sided) was defined as statistically significant

*AF* Atrial fibrillation, *BMI* body mass index, *HT* hypertension, *DM* diabetes mellitus, *CHD* coronary heart disease, *SBP* systolic blood pressure, *DBP* diastolic blood pressure, *ALT* alanine aminotransferase, *AST* aspartate aminotransferase, *Scr* serum creatinine, *TC* total cholesterol, *TG* triglycerides, *HDL-C* high-density lipoprotein cholesterol, *LDL-C* low-density lipoprotein cholesterol, *FPG* fasting plasma glucose, *TyG* triglyceride-glucose index

### LASSO regression screening results

**Fig. 2:**
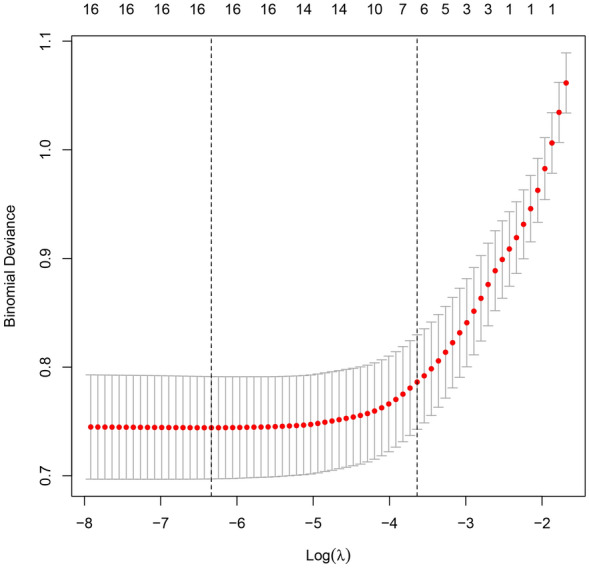
Regularization parameter λ screening process

**Fig. 3:**
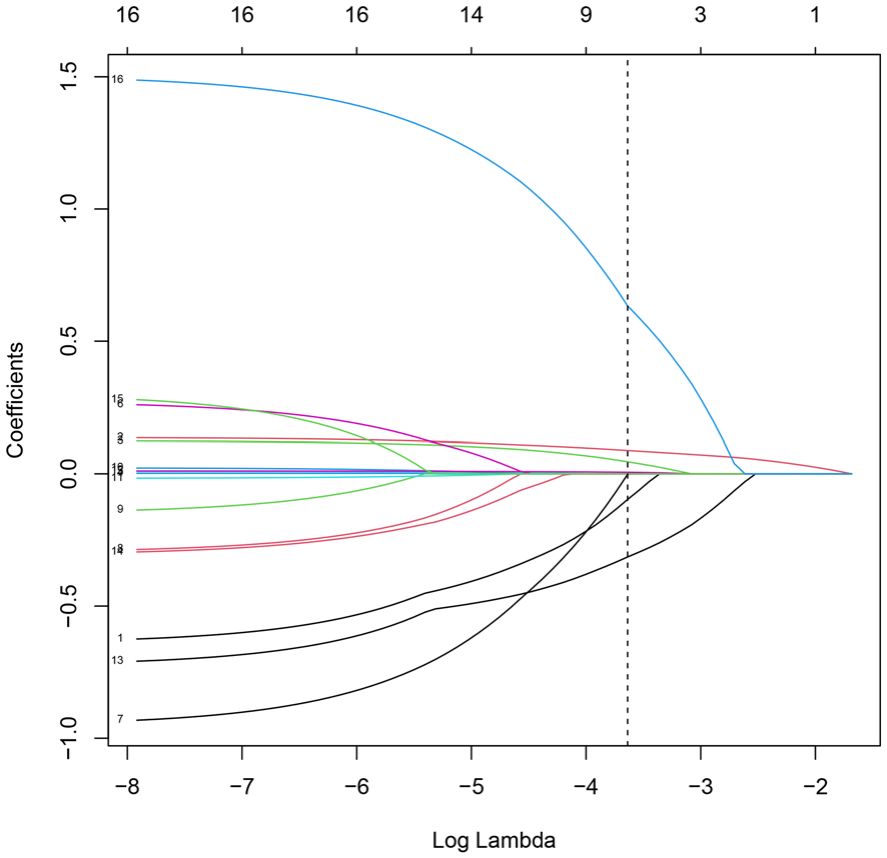
Changes in coefficients for 20 variables when the regularization parameter λ changes

Based on cross-validation, when λ = 0.026, the results of screening AF-related indicators with non-zero coefficients from the above 20 item variables through the LASSO regression algorithm were: gender, age, BMI, DM, Scr, TC, TyG (Additional file 1: Table S1; Figs. 2, 3).

### Univariate and multivariate analysis of AF and TyG in NAFLD

**Fig. 4:**
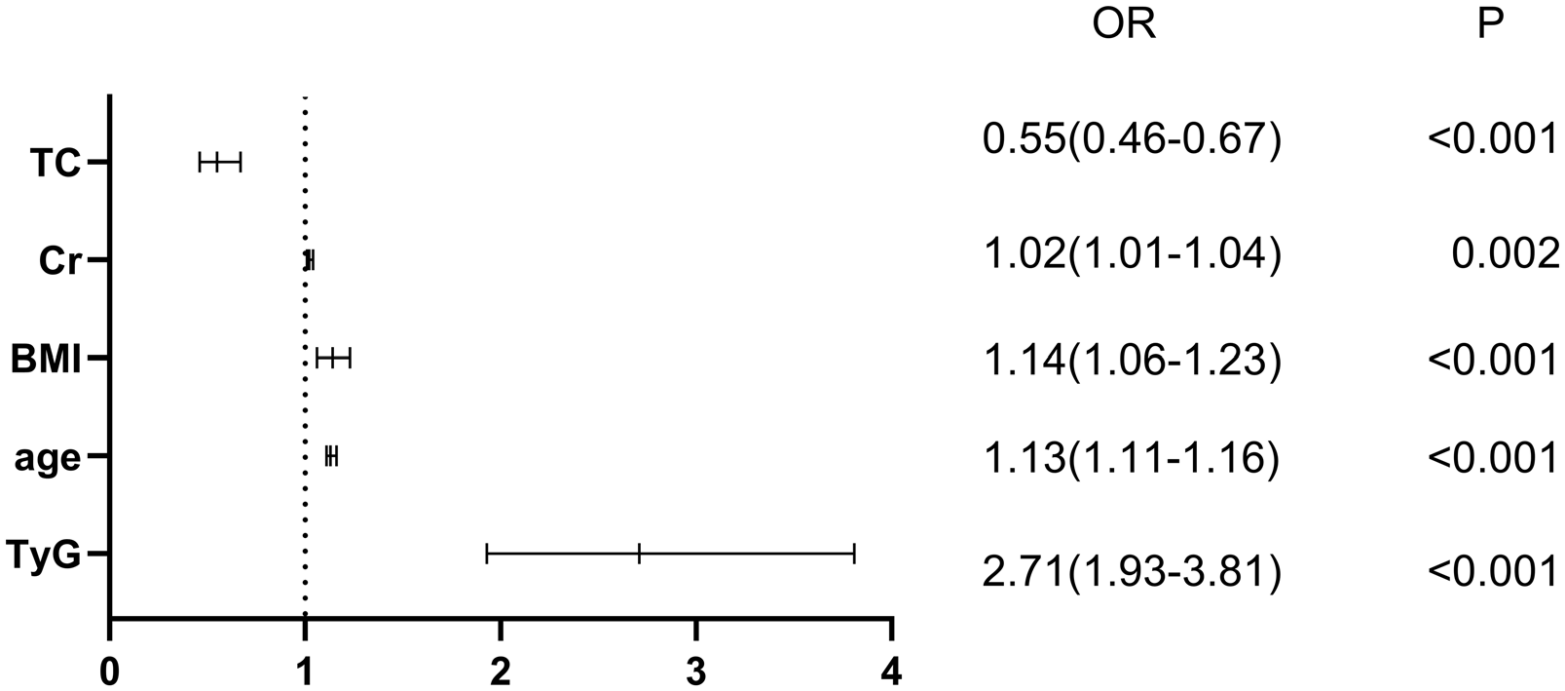
Forest plots of independent factors associated with atrial fibrillation (AF) in non-alcoholic fatty liver disease (NAFLD)

The multivariate LASSO logistic regression analysis showed that TyG was significantly correlated with the prevalence of AF (Table 2). In the absence of adjustments, each unit increase in TyG correlated with a 2.71-fold increase in the risk of AF (odds ratio [OR] = 2.71, 95% confidence interval [CI] 1.93–3.81, P < 0.001). After adjusting for variables screened by the LASSO regression algorithm (gender, age, BMI, DM, Scr, TC), a one-unit increase in TyG increased the risk of AF by 4.84 (OR = 4.84, 95% CI 2.98–7.88, P < 0.001). In addition, the study identified that the correlation between TyG and AF was significantly stronger than that of other clinical variables (Fig. 4; Additional file 1: Table S2).

**Table 2:** LASSO logistic regression assesses the correlation between TyG and the prevalence of atrial fibrillation

	No-adjust		Lasso logistic	
	OR (95%CI)	P	OR (95%CI)	P
TyG	2.71 (1.93–3.81)	< 0.001	4.84 (2.98–7.88)	< 0.001
TyG 1	Ref.		Ref.	
TyG 2	1.00 (0.61–1.64)	1.000	1.20 (0.66–2.17)	0.553
TyG 3	1.48 (0.93–2.35)	0.101	1.93 (1.07–3.49)	0.030
TyG 4	2.50 (1.60–3.90)	< 0.001	4.34 (2.37–7.94)	< 0.001
P for trend	P < 0.001		P < 0.001	

Lasso Logistic was adjusted for age, sex, DM, BMI, TC and Scr

To understand the relationship between TyG levels and AF in patients with NAFLD, TyG levels were grouped into quartiles. The average TyG index of the four groups were 8.39 ± 0.20, 8.78 ± 0.83, 9.09 ± 0.99 and 9.57 ± 0.27. Statistically significant differences were noted among the four groups in the rates of AF, age, HT, DM, CHD, SBP, ALT, AST, TC, TG, HDL-C, LDL-C, utilization rate of antidiabetic drugs and FBG (all P < 0.05) (Table 3).

**Table 3:** Demographic and clinical characteristics of participants by TyG

	TyG 1	TyG 2	TyG 3	TyG 4	P
Age (years)	58.11 ± 10.94	57.74 ± 11.55	60.03 ± 11.86	60.34 ± 12.37	0.034
Gender (male)	139 (61.0)	142 (62.3)	138 (60.5)	142 (62.3)	0.972
Smoke [(n%)]	91 (39.9)	92 (40.4)	107 (46.9)	91 (39.9)	0.343
HT [(n%)]	120 (52.6)	123 (53.9)	141 (61.8)	147 (64.5)	0.023
DM [(n%)]	22 (9.6)	50 (21.9)	76 (33.3)	117 (51.3)	< 0.001
CHD [(n%)]	103 (45.2)	112 (49.1)	120 (52.6)	138 (60.5)	0.009
BMI (kg/m^2^)	25.91 ± 2.65	25.98 ± 2.90	25.85 ± 2.76	26.35 ± 2.90	0.228
SBP (mmHg)	129.89 ± 16.54	132.12 ± 18.32	134.89 ± 19.97	131.41 ± 20.07	0.037
DBP (mmHg)	79.93 ± 12.83	80.60 ± 12.78	80.33 ± 13.56	79.20 ± 13.15	0.688
ALT (U/L)	21.80 (16.00,29.70)	22.55 (16.20,30.68)	26.85 (18.25,34.15)	26.05 (18.70,34.50)	< 0.001
AST (U/L)	21.50 (18.00, 26.10)	22.50 (18.40, 28.30)	23.30 (18.50, 29.28)	23.40 (18.30, 30.60)	0.039
Scr (mmol/L)	67.00 (58.00, 77.00)	67.50 (59.00, 77.00)	65.18 (58.82, 75.92)	68.00 (56.25, 78.00)	0.699
TC (mmol/L)	3.87 (3.26, 4.73)	4.13 (3.57, 5.01)	4.49 (3.64, 5.35)	4.58 (3.75, 5.41)	< 0.001
TG (mmol/L)	1.13 (0.98, 1.27)	1.48 (1.29, 1.71)	1.94 (1.65, 2.20)	2.53 (2.02, 2.98)	< 0.001
HDL-C (mmol/L)	1.19 (1.00, 1.40)	1.08 (0.92, 1.29)	1.05 (0.95, 1.20)	1.02 (0.88, 1.17)	< 0.001
LDL-C (mmol/L)	2.10 (1.56, 2.60)	2.28 (1.89, 2.75)	2.40 (1.87, 2.92)	2.42 (1.85, 2.92)	< 0.001
FBG (mmol/L)	5.06 (4.63, 5.45)	5.39 (4.85, 6.25)	5.72 (5.16,6.90)	7.15 (5.81, 8.59)	< 0.001
Anti-diabetic drugs[(n%)]	16 (7.0)	39 (17.1)	72 (31.6)	109 (47.8)	< 0.001
Lipid-lowering drugs[(n%)]	58 (25.4)	65 (28.5)	73 (32.0)	82 (36.0)	0.084
TyG	8.39 ± 0.20	8.78 ± 0.83	9.09 ± 0.99	9.57 ± 0.27	< 0.001
AF [(n%)]	38 (16.7)	38 (16.7)	520 (22.8)	76 (33.3)	< 0.001

Values are expressed as mean ± standard deviation, no. (%), or median (interquartile range). P < 0.05 (two-sided) was defined as statistically significant

**Fig. 5:**
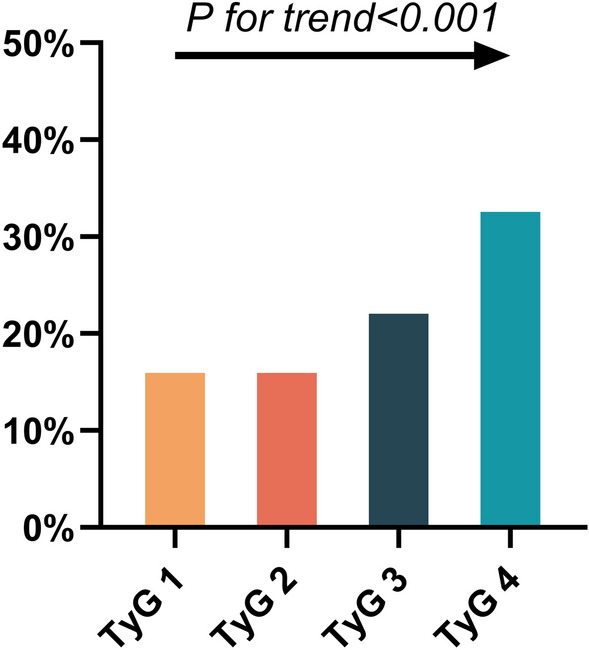
Prevalence of atrial fibrillation (AF) in patients with non-alcoholic fatty liver disease (NAFLD) at different quartiles of triglycerides-glucose index (TyG)

**Fig. 6:**
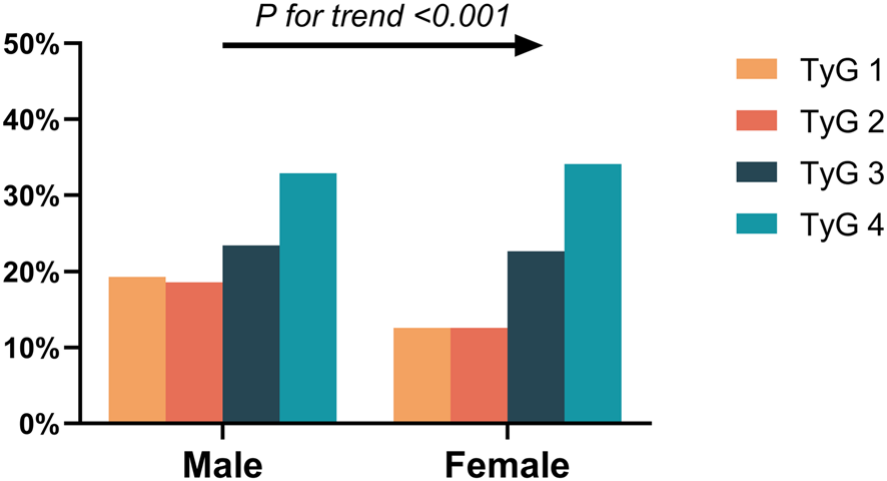
Incidence of atrial fibrillation (AF) based on the sex-subgroup triglycerides-glucose index (TyG) index quartile

For further logistic analysis, the TyG level in the first quartile was set as the control group. The LASSO logistic regression algorithm showed that even after full adjustment, the third and fourth risk of AF remained 1.93 and 4.34 times that of the first quartile (OR = 1.93, 95% CI 1.07–3.49, P = 0.030), (OR = 4.34, 95% CI 2.37–7.94, P < 0.001). However, the OR value for the second quartile was not statistically significant (OR = 1.20, 95% CI 0.66–2.17, P = 0.553). Second, the trend test showed a a trend in AF incidence between TyG quartiles (P for trend < 0.001) (Fig. 5). In conclusion, increased TyG levels correlated with an increased risk for AF. This correlation persisted in the subgroup analysis by sex (P for trend < 0.001) (Fig. 6).

**Fig. 7:**
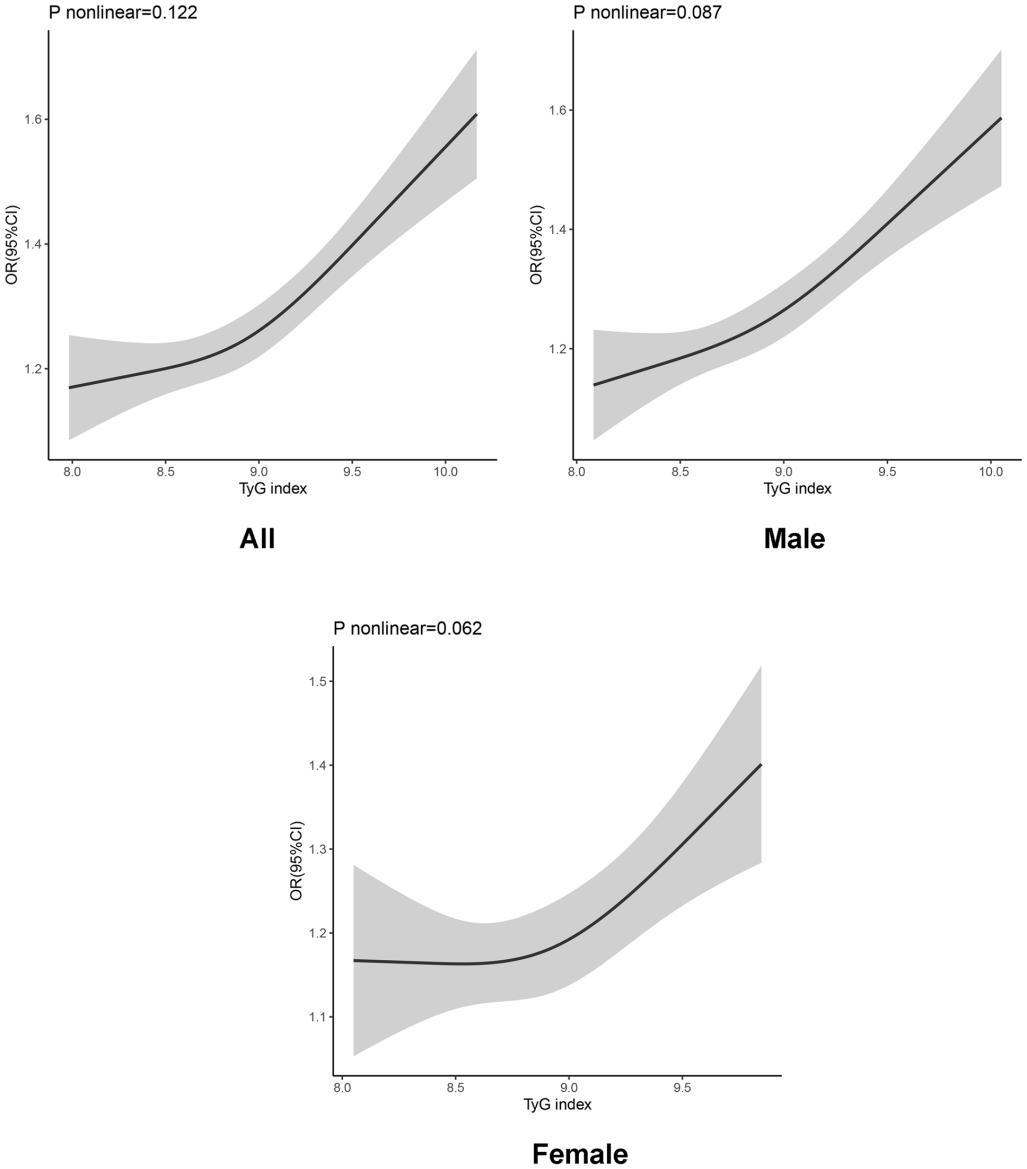
Association between triglycerides-glucose index (TyG) index and atrial fibrillation (AF) was shown using restricted cubic splines (RCS) with three segments of the 10th, 50th and 90th percentiles of the TyG index, adjusting for age, sex, body mass index (BMI), Diabetes mellitus (DM), total cholesterol (TC), and serum creatinine (Scr)

Our study further used RCS to prove the correlation between TyG and AF, and the results are shown in Fig. 7. There was a linear increase in the risk for AF with increasing TyG levels across the entire TyG range. Furthermore, the log-likelihood test demonstrated significant linearity (P nonlinear = 0.122). After grouping by sex, this linear trend was consistent.

### Subgroup analysis of the correlation between the TyG index and AF

**Fig. 8:**
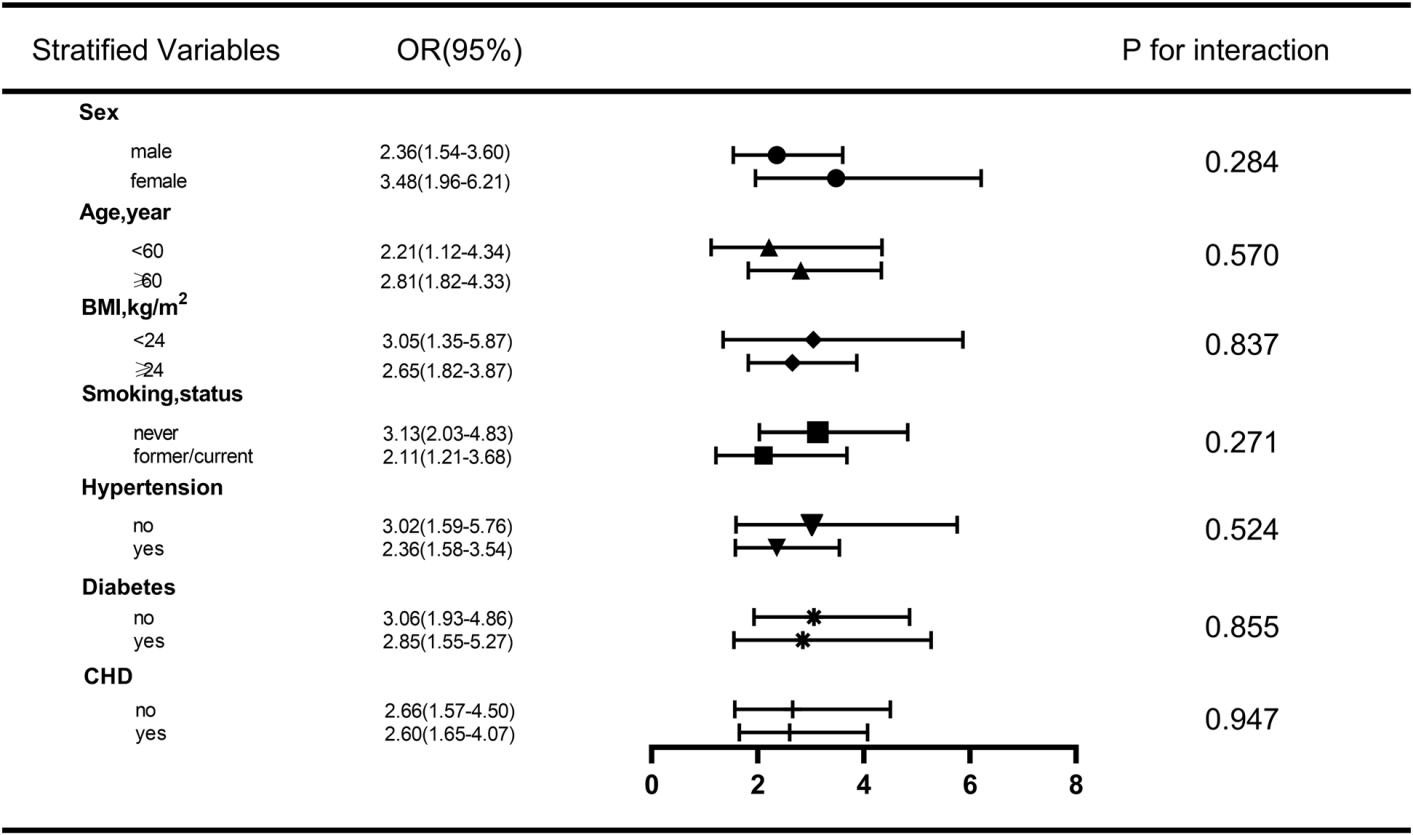
Subgroup analysis for the association between triglycerides-glucose index (TyG) and atrial fibrillation (AF)

We performed several stratified analyses to further assess the robustness of the relationship between the TyG index and AF. As shown in Fig. 8, after grouping according to sex, age, BMI, smoking, HTN, DM, and CHD, TyG was associated with a high AF risk in all subgroups, and no interaction was found in any of the subgroup analyses (P for interaction > 0.05).

### ROC analysis of the TyG index

**Fig. 9:**
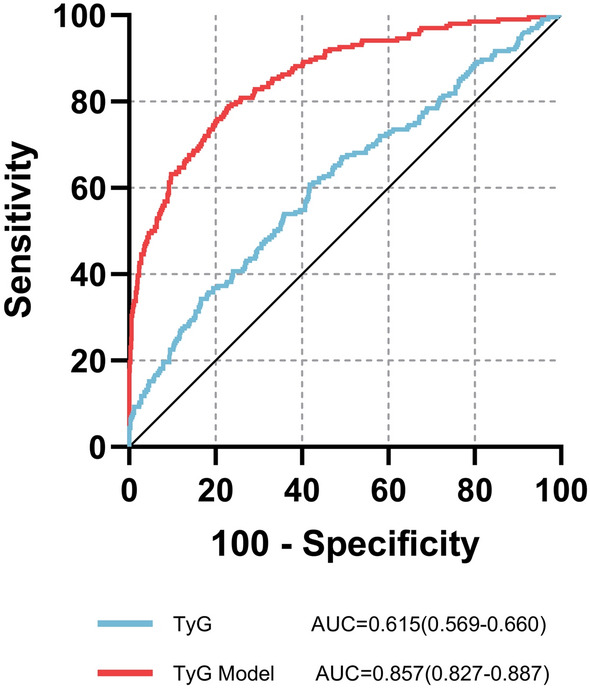
Receiver operating characteristic (ROC) curve analysis of the predictive power of triglycerides-glucose index (TyG) for atrial fibrillation (AF). TyG Model The new model integrates risk factors for LASSO regression screening (sex, age, body mass index [BMI], Diabetes mellitus [DM], total cholesterol [TC], and serum creatinine [Scr])

ROC analysis between TyG and the incidence of AF evaluated the usefulness of TyG in predicting AF in patients with NAFLD. The area under the curve (AUC) for only TyG was 0.615 (95% CI 0.569–0.660, P < 0.001). After introducing LASSO regression screening factors (including age, sex, BMI, DM, TC, and Cr), the ability of TyG to predict AF significantly improved, and an AUC of 0.857 (95% CI 0.827–0.887, P < 0.001) was observed (Fig. 9).

## Discussion

NAFLD is a public health problem that affects approximately one-third of the adult population [18]. Studies have shown that the clinical burden of NAFLD includes liver-related morbidity and mortality. In addition, evidence showing that NAFLD is a multisystem disease that confers a high risk of cardiovascular disease independent of traditional risk factors is increasing [19]. Importantly, the multisystem nature of NAFLD correlates with nonischemic heart disease, particularly changes in cardiac electrophysiology. AF is the most common type of chronic cardiac arrhythmia worldwide. Since life expectancy is increasing, the lifetime incidence of AF is simultaneously increasing. An increasing incidence of AF correlates with a higher risk of embolic stroke and heart failure [20, 21]. Recent epidemiological evidence has shown that NAFLD increases the risk of cardiovascular disease and AF [22]. In a cohort study of 334 280 individuals, Roh et al. [23] found that NAFLD assessed using the fatty liver index independently correlated with an increased risk of new-onset AF in the healthy Korean population. In addition, the results of this study confirm that NAFLD can lead to AF in the absence of intermediate events such as DM, HTN, heart failure, or myocardial infarction. A large-scale observational study [3] that included patients from national examination centers in China in 2022 confirmed that NAFLD correlates with a significantly higher risk of AF in a cross-sectional population and longitudinal cohort. In a meta-analysis [24] of 19 studies involving 7,012,960 patients, NAFLD was independently associated with a higher risk of AF.

As mentioned above, although the presence of NAFLD increases the risk of AF, the underlying mechanism for this correlation is unknown. Dyslipidemia, IR, an inflammatory environment, and the renin-angiotensin system activation are currently considered pathophysiological mechanisms linking AF and NAFLD [7].

NAFLD is defined as excessive fat accumulation in the liver after an imbalance in lipid acquisition and consumption when the liver's metabolic capacity is overwhelmed [25]. IR is an integral part of NAFLD pathogenesis and is closely related to hepatic lipid metabolism pathways [26]. However, evidence shows that IR caused by NAFLD does not only occur in the liver. Furthermore, this IR strongly correlates with non-hepatic tissue IR and is unrelated to obesity [25, 27]. The liver is the center of systemic lipid and glucose metabolism. In addition, the liver synthesizes and releases various metabolites that transmit metabolic signals that potentially cause systemic IR [3].

In contrast, elevated fetuin-A and selenoprotein P levels have been observed in patients with NAFLD. These molecules may cause systemic IR by directly targeting insulin signaling and indirectly affecting glucose and lipid metabolism [28, 29]. This systemic IR state can cause excessive accumulation of fatty acids and triglycerides in cardiomyocytes. This accumulation results in a phenomenon known as “cardiac lipotoxicity.” Cardiac lipotoxicity induces cellular dysfunction, cardiomyocyte apoptosis and impairs myocardial metabolism. These changes may lead to cardiomyocyte function and structure alterations and thus increase the risk of arrhythmias [30–32].

Additionally, an association between IR and AF was reported in animal experiments. Studies have shown that IR causes atrial structural remodeling and abnormal intracellular calcium homeostasis, thereby increasing the likelihood of AF [33]. Animal studies have also shown that IR induces the impaired transport and expression of the major cardiac subtype glucose transporter type (GLUT)4 and the novel subtype GLUT8. This increases the vulnerability and propensity for spontaneous AF [34]. Clinical studies have also demonstrated a correlation between IR and AF. Wang et al. [35] found that patients with IR had an increased incidence of recurring AF after radiofrequency catheter ablation. Lee et al. [8] found a strong correlation between IR and AF in patients without diabetes during a median follow-up of 12.3 years. Moreover, a cohort study and in vitro experiments [36] found that using the insulin sensitizer metformin reduced the risk of AF in patients with type 2 diabetes. Therefore, metformin might attenuate tachycardia-induced myolysis and oxidative stress in atrial cells.

The TyG index is a simple and reliable surrogate marker for IR. TyG has a high sensitivity (96.5%) and specificity (85.0%) in the diagnosis of IR compared to normoglycemic hyperinsulinemic clamp. Therefore, TyG may be widely used as a marker for IR in clinical practice [37]. Numerous studies have reported that the TyG index reflects the state of IR in the body and correlates with inflammation, metabolic disorders, abnormal coagulation function, and thrombosis [38–40]. Studies have also revealed the association between the TyG index and AF incidence. Shi et al. [41] found a significant linear association between the TyG index and AF incidence in a diabetic population. Ling et al. [42] demonstrated that the TyG index was an independent predictor of new-onset AF after percutaneous coronary intervention (PCI), followed by Wei et al. [43] in 409 cases of hypertrophic obstructive myocardium undergoing septal muscle resection A significant association was found between TyG levels and the risk of postoperative AF in these patients. Although it is an effective predictor of NAFLD [44], its relationship with AF in patients with NAFLD remains unclear.

Our results showed a significant correlation between TyG and AF levels in the NAFLD population. In addition, this study found that age and BMI in the AF group were significantly higher than those in the non-AF group. This indicates that age and elevated BMI may increase the risk of atrial fibrillation, which is consistent with previous studies [45]. In addition, baseline data showed that BMI was higher than normal in both the AF and non-AF groups, which may be related to NAFLD and systemic IR. Previous studies [46] showed that obesity is an independent risk factor for NAFLD, which correlates with an increased prevalence and severity of NAFLD. However, NAFLD has been diagnosed in patients with normal BMIs [47]. The result of the TyG index might vary depending on the BMI. Therefore, we performed a subgroup analysis based on BMI, which showed that TyG index was significantly associated with the prevalence of atrial fibrillation even in non-obese subjects (BMI < 24 kg/m2). Overall, these results indicate the potential of the TyG index as a predictor of AF in patients with NAFLD.

In contrast, a meta-analysis [48] showed that serum TC, LDL-C, and HDL-C levels were negatively associated with AF risk, but there was no significant association between TG levels and event AF. These results were consistent with experimental results.

Logistic regression is the most widely used regression model in medical research. Multicollinearity may lead to fluctuations in the regression results and poor model stability [49]. To solve this, variables may be added or subtracted using stepwise regression to obtain a valid set of predictive variables. However, stepwise regression cannot completely overcome severe multicollinearity in the model. LASSO regression effectively addresses the issue of multicollinearity in the model. It compresses unimportant regression coefficients to zero by introducing a penalty term into the model. Therefore, higher prediction accuracy and generalization abilities are obtained at the cost of an estimation bias [50]. In the AF-related regression model, including all variables in the model may lead to a correlation between each variable of the linear model and influence the stability of the model and the estimated distortion. Therefore, we introduce the LASSO regression model that filters variables, selects representative variables, and various related prediction models to establish the correlation with AF and reduce the model’s instability and estimation distortion caused by multicollinearity.

A large sex difference in AF was noted. The mechanisms, etiology, response to treatment, stroke risk, and outcomes differed significantly between the sexes [51]. Therefore, in further studies, the impact of sex on the influence of TyG on AF incidence should be assessed. However, our results showed that the risk of AF increased linearly with an increase in TyG over the entire TyG range. When grouped by sex, the trend remained unchanged.

This study had several limitations. First, this study was a cross-sectional study with a small sample; The low prevalence of atrial fibrillation among study participants limits the extrapolation of current results to different types of atrial fibrillation, and a single center and small sample size may lead to bias; Second, we relied on a single ECG and patient self-report for the definition of AF. Therefore, the accuracy of the AF diagnosis may have been affected. Third, we did not differentiate between paroxysmal and persistent AF. Whether there is a difference in the relationship between TyG levels and the incidence of different AF types requires further investigation. Fourth, similar to other observational epidemiological studies, residual confounding caused by unincluded covariates may have biased the results. For example, our variables did not include information on electrolyte disturbances or medications that increase the risk of AF. Finally, because this was a cross-sectional study, the results showed that the TyG index was positively associated with AF in patients with NAFLD but could not claim predictive value. Future large-scale multicenter prospective studies are required to verify the predictive power of the TyG index for the risk of AF in patients with NAFLD.

## Conclusion

The results showed that TyG levels in NAFLD patients with AF were significantly higher than in non-AF patients with AF. TyG level is a simple and easy-to-obtain parameter that can be used in clinical practice to predict the risk of AF in patients with NAFLD. Therefore, this indicator should be assessed in managing patients with NAFLD.

## Supplementary Information

**Additional file 1.** Additional tables.

## Data Availability

The original contributions of this study are included in the article/supplementary material. Further inquiries can be directed to the corresponding author.

## Abbreviations

AF: Atrial fibrillation
ALT: Alanine aminotransferase
AST: Aspartate + aminotransferase
CHD: Coronary heart disease
CVD: Cardiovascular disease
DBP: Diastolic blood pressure
DM: Diabetes mellitus
FBG: Fasting blood glucose
HDL-C: High density lipoprotein cholesterol
HT: Hypertension
IR: Insulin resistance
LDL-C: Low-density lipoprotein cholesterol
NAFLD: Non-alcoholic fatty liver disease
Scr: Serum creatinine
TC: Total cholesterol
TG: Triglyceride
TyG: Triglycerides-glucose index
