# Supplementary material for: Correlation between the triglyceride-glucose index and the onset of atrial fibrillation in patients with non-alcoholic fatty liver disease
Source: Diabetol Metab Syndr. 2023 May 9;15:94. doi: 10.1186/s13098-023-01012-1 (PMC10169476; doi:10.1186/s13098-023-01012-1)
Supplement: Supplementary file 1 — Additional file 1. Additional tables. [file 13098_2023_1012_MOESM1_ESM.docx]

Table S1 Test index with non-zero coefficient and its Coef value

| Variables | Coef |
| --- | --- |
| Gender | -0.097 |
| Age | 0.088 |
| BMI | 0.046 |
| Diabetes | -0.002 |
| Cr | 0.006 |
| TC | -0.314 |
| TyG | 0.634 |

Table S2 LASSO logistic regression assesses the correlation between other variables and the prevalence of atrial fibrillation.

|  | Lasso Logistic | |
| --- | --- | --- |
|  | OR(95%CI) | P |
| Age | 1.13(1.11-1.16) | <0.001 |
| BMI | 1.14(1.06-1.23) | <0.001 |
| Scr | 1.02(1.01-1.04) | 0.002 |
| TC | 0.55(0.46-0.67) | <0.001 |

Table S3 Correlation between triglyceride-glucose index (TyG) and atrial fibrillation (AF) at different BMI levels

|  |  | P | OR(95%CI) |
| --- | --- | --- | --- |
| BMI | <24 | 3.05(1.35-5.87) | 0.007 |
|  | ≥24 | 2.65(1.82-4.33) | <0.001 |

adjusted for age, sex, DM，TC and Scr.
